# Development and Application of Carbonate Dissolution Test Equipment under Thermal–Hydraulic–Chemical Coupling Condition

**DOI:** 10.3390/ma15207383

**Published:** 2022-10-21

**Authors:** Jinzhu Meng, Sili Chen, Junxiang Wang, Zhi Chen, Jingyu Zhang

**Affiliations:** School of Architecture and Civil Engineering, Shenyang University of Technology, Shenyang 110870, China

**Keywords:** carbonate rock, thermal–hydraulic–chemical coupling, dissolution test equipment, dissolution effect

## Abstract

The latest continuous flow micro reaction technology was adopted to independently develop carbonate rock dissolution test equipment. Carbonate rock dissolution tests were conducted under different temperatures, flow rates, and dynamic water pressure conditions to study the dissolution process of carbonate rocks under the coupling of heat-water-chemistry. The dissolution effect and development law of carbonate rocks were explored by quantitatively studying carbonate rock dissolution rate and chemical composition of karst water. The results showed that the self-designed dissolution test equipment has obvious advantages. After dissolution, carbonate rock specimens were damaged to varying degrees. The dissolution rate was proportional to water velocity and hydrodynamic pressure, with the velocity effect being greater than the hydrodynamic pressure effect. The pH value, conductivity, and Ca^2^^+^ ion content of the reaction solution gradually increased after dissolution. The development and application of the equipment have proved that, at low dynamic water pressures (2 MPa), the water flow velocity effect on the dissolution velocity was 1.5 times that when the dynamic water pressure was high (6 MPa); at a low water flow velocity of 15 mL/min, the dynamic water pressure effect on the dissolution velocity was three times that when the water flow velocity was high (75 mL/min). The development process is gradually becoming strong and stable. Its research has important theoretical significance and engineering application value to provide technical means and guarantee for the early identification, karst development, and safety evaluation of karst geological disasters.

## 1. Introduction

The lithology of China’s karst stratum is predominantly limestone, and the geological conditions are complex. The soluble rock distribution area accounts for more than one-third of the land area, making it one of the countries with the world’s largest karst distribution areas and karst development types. Deep resource extraction, energy extraction, and urban underground space development are just a few of the underground initiatives that are becoming more prevalent as the social economy expands. Karst slopes, foundation stability, karst water gushing, mud outburst, and karst collapse negatively affect structure foundations and pose significant safety and investment risks to engineering construction. So, to carry out engineering construction in karst areas and to improve the stability of engineering structures and later safety maintenance, it is necessary to understand the characteristics of rock dissolution law in soluble rock areas, predict its development trend, and provide assurance for early identification and safety evaluation of karst geological disasters. Europe, the United States, and other countries and regions have carried out large-scale karst landforms [1,2], hydrogeology [3,4], karst environment [5,6], and other aspects to further develop karst research.

The research methods can be divided into dissolution mechanism theory research and karst detection research. Dissolution tests are one of the most significant contents and methods for researching the dissolution mechanism, which is a significant part of theoretical research to investigate further the controlling factors and impacts of carbonate rock dissolution. Since the 1970s, scholars have conducted experimental research on carbonate rock dissolution under thermal, hydraulic, and chemical factors. Eisenlohr et al. [7], Sjoberg et al. [8], Amrhein et al. [9], Buhmann et al. [10], and Ma et al. [11] systematically studied carbonate rock dissolution and precipitation and summarized the kinetic factors. Plumer et al. [12,13] further found the calcite surface dissolution reaction law in saturated CO_2_ water solution under different test conditions and its dynamic process. Alkattan et al. [14], Pokrovsky et al. [15], and Sjoberg et al. [16] studied the dissolution rate of rock samples and their influencing factors. The study of a single mineral’s characteristics of dissolution under the control of a single factor in the research has advanced to the simulation of seepage and dissolution in the rock under the joint control of multiple means. Experimental conditions have evolved from early low-temperature, low-pressure conditions [17,18] to high-temperature, high-pressure conditions [19,20,21] and from closed-system, static conditions [22,23] to open-system, flow conditions [24,25,26]. Rock samples from limestone, dolomite, and other different sources [27], different types [28,29,30,31], and different types of seawater, acetic acid, natural water body, and CO_2_ water solutions [30,32,33,34,35] are gradually increasing. This study investigates the effect of various external factors on carbonate rock dissolution by varying the experimental control factors. According to the study’s findings on the effect of temperature on carbonate rock dissolution, some researchers think that normal and medium temperature conditions are preferable to low and high temperatures for carbonate solution to facilitate carbonate rock dissolution [36]. Concerning the impact of flow rate on the dissolution of carbonate rocks, Gao [37] discovered that the dissolution of carbonate rocks by solution under flowing conditions is always greater than that under closed conditions, and Shao’s [38] experiment confirmed that the change in flow rate has a significant impact on the dissolution rate. Robert et al. [19], Yang et al. [39], Shou et al. [40], and Liu et al. [25] investigate the impact of pressure on carbonate rock dissolution and have a consistent understanding of the pressure factor carbonate rock dissolution; that is, carbonate rock dissolution increases with increasing pressure. However, previous tests did not include the coupling of water flow speed and dynamic water pressure, and its dissolution effect and development law. The pH value of carbonic acid solution obviously influences the dissolution rate of rock samples [41,42,43,44], and the dissolution rate increases with decreasing pH value. Zhao et al. [45] established that the dissolution rate in acid is approximately 20–400 times that in alkaline and neutral conditions.

Many scholars performed an experimental study by self-designing experimental equipment, constantly updating and enhancing various control factors of the experiment from the standpoints of solution preparation, pressure system, and hydrodynamic system, and further exploring the dissolution effect of multifactor coupling of carbonate rocks. There are two solution preparation systems: organic acid preparation [46,47] and carbonic acid solution preparation [22]. The most common organic acid in buried diagenetic oilfield water is acetic acid, which is produced from deionized water and pure acetic acid reagent. She et al. [48] and Yang [49] use acetic acid solution. Although it is simple to prepare and produce accurate results, its applicability is only limited. Carbonate solution preparation, the most crucial dissolution medium for carbonate under near-surface supergene conditions, is the other type of solution preparation. This preparation technique is employed by Wang et al. [50], Shao [26], Gao [37], and Fan et al. [51] for their experiments. Gas and liquid must be introduced into a larger container that can be used continuously. It has many flaws although it has a broad range of applications. The instrument is large and difficult to override, the water supply tank is positioned at a high point, and the reaction vessel has a large volume. In earlier testing, the larger vessel’s volume reached 1500 L. However, the long-term dissolution test could not be performed even with such a massive tank. Second, the configuration time is long, and the dissolution reaction time of most experiments is 7 days, with some going as long as 15 days. Even with the magnetic drive stirring device, the reaction will be slow. Third, the accuracy is low, and there are numerous impacting factors in the reaction process, including the absolute pressure of the gas–liquid system, liquid temperature, contact area and time between gas and liquid, and so on. The flow rates of water and CO_2_ must be constantly adjusted, and adequacy must be judged by the numerical value of the liquid surface pressure gauge to achieve the required pH value. Because the entire process is based on experience and repeated attempts, judging the gas–liquid reaction’s adequacy and the test results’ accuracy is impossible. The pressure system in the equipment is primarily pressurized by nitrogen and is actuated by a high-pressure pump and a back-pressure valve: nitrogen pressurization means that a high-pressure N_2_ gas tank provides a specific pressure value. For example, Liu et al. [25] formed a pressurizing and stabilizing system by sealing a high-pressure water tank, an airbag, and a pressure reducer with an automatic compensation function, which can meet the constant pressure test to exclude the influence of pressure change factors, such as constant pressure and variable flow rate. Another method of pressurization is the use of a high-pressure pump and a back-pressure valve, with the pressure supplied by the high-pressure pump driving device and stabilized by the back-pressure valve controller. Although Fan et al. [51], Jiang et al. [52], and She et al. [53] achieved pressure value regulation using this method, However, the test did not perform the simultaneous regulation of the hydrodynamic system, that is, the pressure is variable while the flow rate is constant. The test equipment’s hydrodynamic system can simulate two types of water flow state: static erosion and osmotic erosion. Contact erosion occurs when there is no water head or hydraulic gradient. For example, Zhao et al. [45] and Huang and Song [54] used the direct immersion method to perform static erosion, and while the test procedure was simple, the actual water flow state could not be simulated. The other flow state is seepage erosion with water head or hydraulic gradient, which is classified into two types based on whether or not the flow rate changes. Fan et al. [36,51] and Jiang et al. achieve the fixed flow rate. Jiang et al. [52] use the methods of fixed time, fixed flow rate, and fixed total amount. She et al. [53] and Gao et al. [37] employed a liquid pump to drive the flow rate, leading to a constant flow rate. Su [55] and Shao [26] applied a liquid pump to control the flow rate, which enabled them to observe the impact of temperature on the dissolution rate of carbonate rocks at different flow rates. However, they did not include the pressure factor, such as the flow rate.

Currently, most research on carbonate rock erosion is based on a single environmental equilibrium state with less emphasis on nonequilibrium state systems in different occurrence environments, focusing on the relationship between control factors, such as temperature, velocity, pressure, and the erosion effect. Because the test solution preparation process is complicated, the reaction time is long, and the accuracy is low, the hydrodynamic and pressure systems cannot be controlled simultaneously. In this paper, the experimental equipment for carbonate rock dissolution under dynamic water pressure is developed, and dynamic dissolution tests of carbonate rock under different flow rates and pressures are performed to address the problems of carbonated water preparation and parameter setting of the test system. The development trend of karst under thermal–hydraulic–chemical coupling conditions is predicted, and previous experimental equipment limitations are overcome. More water flow rates and pressures can be simulated in the future, allowing for a more comprehensive study of carbonate rock’s hydrochemical kinetic parameters and dissolution characteristics.

## 2. Test Equipment and Apparatus

**Figure 1:**
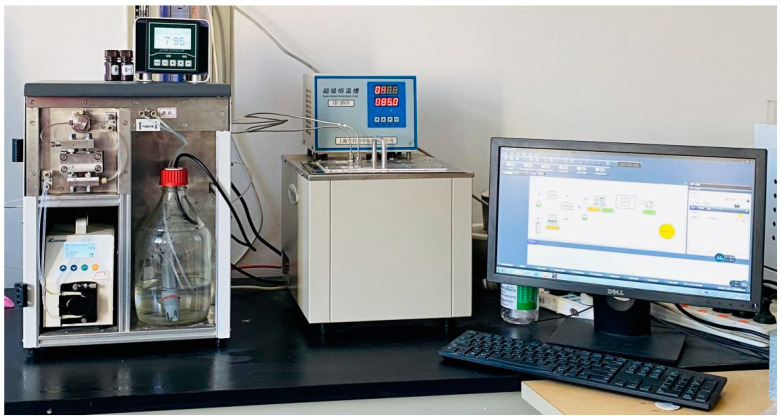
Self-made YDYR-2 rock hydrodynamic pressure dissolution test equipment.

**Figure 2:**
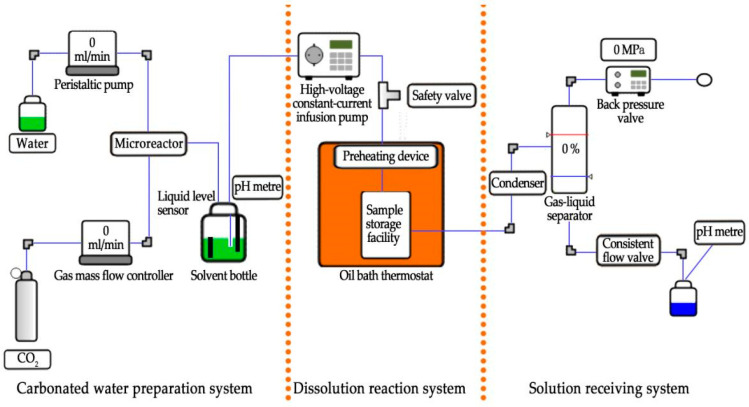
Schematic diagram of the composition of the dissolution test equipment.

A set of YYDR-2 rock hydrodynamic pressure dissolution test equipment (Figure 1) based on microreaction technology was created using the most recent continuous flow microreaction technology. The test equipment consists of three systems: carbonated water preparation, dissolution reaction, and dissolution reception (Figure 2). All of them are controlled and set by computer system control software. The instrument is composed of 316-L stainless steel. The following are the main technical parameters: the high-pressure constant-current infusion pump has a flow rate control range of 0.1–100 mL/min, a pressure of 10 MPa, and an accuracy of 1%. The flow range of the gas flow controller is 5–1000 sccm, the pressure is 10 MPa, and the accuracy is 0.1%. The back-pressure valve has a back-pressure range of 0.2–10 MPa; the gas separator has a pressure range of 10 MPa. The pH measuring range is 2–14, and the precision is 0.02. The temperature control range of the constant temperature water bath box is room temperature +5–200 °C, with a 0.1 °C accuracy.

### 2.1. Carbonated Water Preparation System

It is developed a carbonated water preparation system for real-time online configuration of solutions with different concentrations, which consists of four parts: liquid supply, gas supply, gas–liquid mixing, and storage: The liquid is extracted using a peristaltic pump, which extracts and precisely controls the liquid supply. The carbon dioxide gas is supplied by a carbon dioxide cylinder (with a two-stage pressure-reducing valve), and the flow value is precisely controlled in real time by a gas mass flow controller. The liquid is mixed with CO_2_ gas using the most recent continuous flow microreaction technology. The microchannel is used to carry out a gas–liquid intensified reaction to ensure rapid and sufficient mixing of gas and liquid, resulting in a carbonic acid aqueous solution with a specific pH value and stable solubility. After the gas–liquid reaction, the final 1-L solvent bottle is used as a carbonic acid aqueous solution container. The built-in pH probe and liquid level sensor can accurately monitor the pH value of the solution at any time. The preparation system can achieve a sufficient and rapid gas–liquid reaction while ensuring a steady carbonic acid aqueous solution supply.

### 2.2. Dissolution Reaction System

The dissolution reaction system comprises a pressure and hydrodynamic device, a temperature control device, and a sample reaction device. For example, the pressure and hydrodynamic control device raises and stabilizes the liquid pressure via the back-pressure valve and adjusts the flow rate via the high-pressure constant-current infusion pump to ensure that the solution can complete the flow rate adjustment at different pressures. Afterward, the solution flows into the sample reaction device, allowing four sample silos to be simultaneously placed within 13 mm and 25 mm. Next, the liquid enters through the upper cover’s liquid inlet, is evenly separated into four parts by the umbrella-shaped design at the top of the inlet filter seat cover and then flows into the four main reaction chambers by the similar design at the bottom of the inlet filter seat cover. The liquid is dispersed as it flows in, fully contacting the rock sample. As it flows out, 316-L stainless steel is used in the outlet filter seat cover. Then, the sample bin and preheater are placed in the oil bath thermostat to form a temperature control device, where a 316-L stainless steel coil is used as the preheating device to quickly and accurately preheat the solution delivered by the high-pressure infusion pump to the required temperature of the test, thereby realizing the room temperature −200 °C temperature control, with a larger more extensive temperature control range than the previous water bath.

### 2.3. Solution Receiving System

Solution condensation, gas–liquid separation, decompression and exhaust, and liquid receiving are the four components of the solution receiving system. The condensing unit also uses a 316-L stainless steel coil to achieve condensation by increasing the liquid flow distance, preventing the temperature of the reacted solution from becoming too high and damaging the test device. On the other hand, the condensing unit increases the flow distance between the sample bin and the gas–liquid separator and cools the reacted solution via air cooling. The reacted solution enters the gas–liquid separator, through the condensing device, and the gas and liquid are separated into two paths. The back-pressure valve connects the separated gas to the outlet to adjust and stabilize the fluid’s circulating pressure. The steady flow valve ensures that the separated liquid flows out of the system steadily and smoothly. The receiving container collects the reacted solution and detects additional parameters.

## 3. Materials and Experimental Methodology

Self-developed test equipment was used to select rock samples and dissolved liquid to simulate the carbonate rock dissolution test under multifactor coupling conditions. Then, different test batches were formed based on the test conditions, and the dissolution test was carried out according to specific steps.

### 3.1. Test Sample

#### 3.1.1. Rock Samples

**Figure 3:**
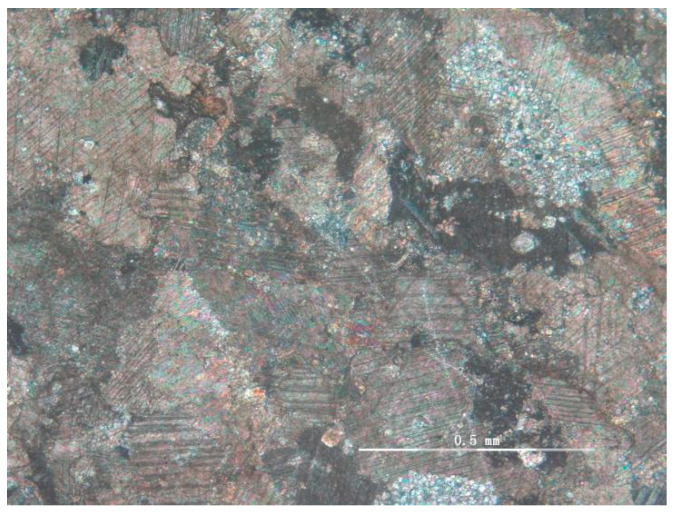
Thin sections of carbonate rocks observed under optical microscopy.

The rock samples used in the experiment were collected from the Dalian Bay coastline. Many karst shapes, such as karst caves, dissolved gaps, and dissolution funnels, were formed in this area due to groundwater and seawater erosion and the influence of structure. Microscopic flakes identified the lithology as crystal grain limestone (Figure 3).

**Figure 4:**
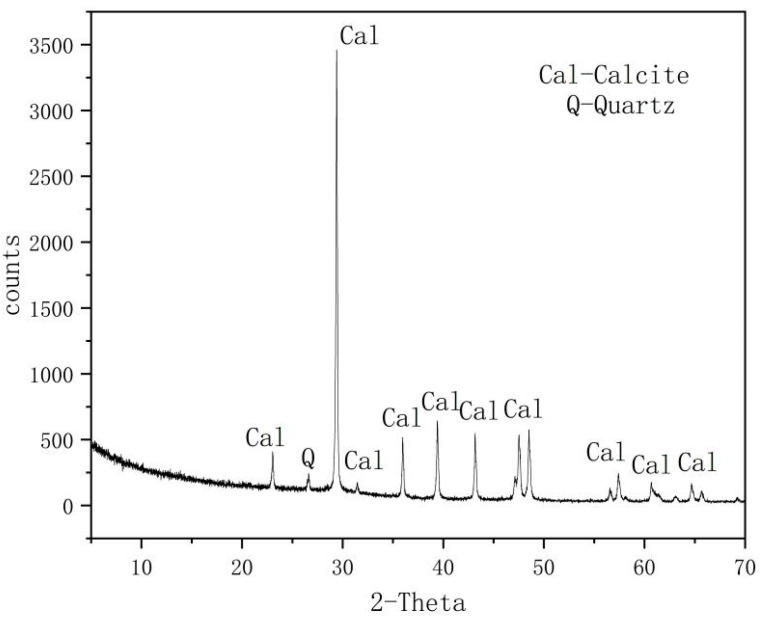
A supplementary figure of the X-ray diffraction whole-rock mineral analysis.

The mineral and chemical composition of rock samples was determined using X-ray diffraction (XRD) and X-ray fluorescence spectrometry (XRF). The mineral composition of the samples tested by XRD analysis is listed in Table 1, and Figure 4 is an attached figure of XRD whole-rock mineral analysis. Calcite was the main mineral in the rock samples, with a trace of Shi Ying. CaCO_3_ is the main component of calcite, and carbonate rock has a total CaO and MgO content > 50%. The chemical composition of the samples obtained by XRF is listed in Table 2. CaO is the main component in the rock samples, with a content of >55.41%, indicating that it is carbonate rock.

**Figure 5:**
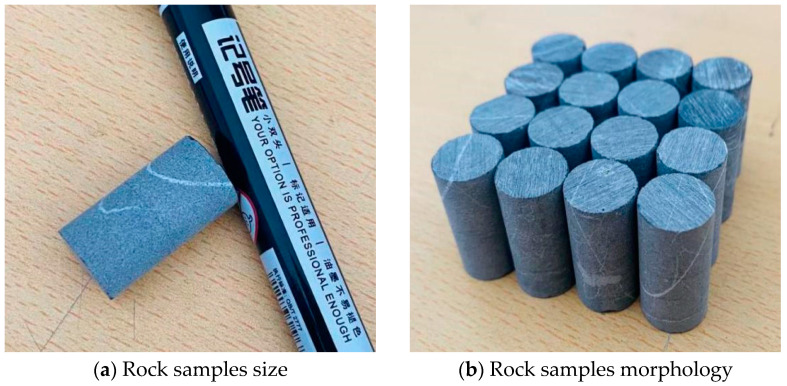
Graphic of rock samples.

Drill a cylindrical rock sample with a core drill with a diameter of 12 mm and a height of 24 mm (Figure 5), wash the rock sample with deionized water, and dry it in a drying box at 105 °C for 12 h. Weigh the test piece W1 before dissolution with an electronic balance with a range of approximately 0–120 g and an accuracy of 0.01 mg.

#### 3.1.2. Sample of Solution

In this experiment, CO_2_ aqueous solution was used as the reaction liquid, and the polar molecule H^+^ was ionized, resulting in carbonate rocks’ strong dissolution. Using tap water as the sample base of the test water is beneficial for detecting the background and ion content after the final test. In addition, it is easy to compare the ion concentration changes. A high-pressure steel cylinder was chosen to provide CO_2_ gas with a certain pressure.

### 3.2. Test Plan

#### 3.2.1. Test Conditions

The self-developed carbonate dissolution test equipment was used in this experiment to quantitatively study the dissolution rate of carbonate rock and the chemical composition of karst water under the thermal–hydraulic–chemical coupling condition, focusing on the coupling study of water flow velocity and pressure. The temperature and pH of the aqueous solution were kept constant to eliminate their influence on dissolution. The goal was to comprehend the dissolution effect of carbonate rock dissolution rate when water flow velocity and pressure were combined.

The following were the test conditions: the solution flow rates, which are 15 mL/min and 75 mL/min, were used to investigate the dissolution effect of drip and line flow. Given the test equipment’s controllable range, two groups of pressure values, 2 MPa and 6 MPa, were chosen to compare the dissolution effects of the two pressure states. The temperature was set to 85 °C, which has a high dissolution ability. The pH of the solution was set to pH = 5, which has a visible dissolution reaction. The working time of the test was set at 7 h in “Physical and Mechanical Properties of Rock Test Specification Part 11: Rock dissolution Test.” Four test batches were created (Table 3).

#### 3.2.2. Test Steps

The rock samples in the sample warehouse were eroded based on the requirements of temperature, hydrodynamic pressure, water flow velocity, and pH value of four groups of test batches. The tests under different conditions were repeated as follows.

**Figure 6:**
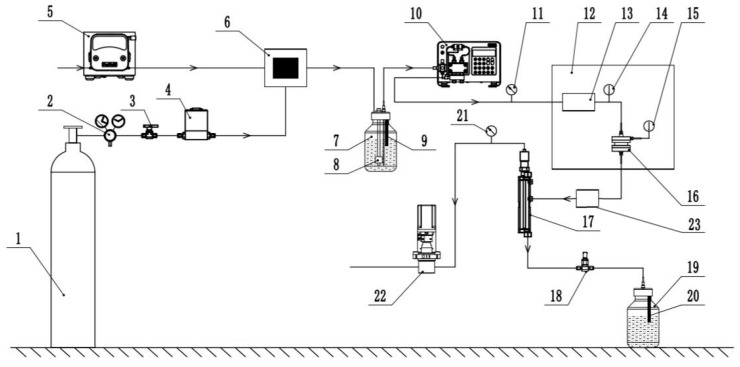
Flow chart of the dissolution test.

Figure 6 components are as follows: 1—CO_2_ high-pressure steel cylinder; 2—two-stage pressure-reducing valve; 3—two-way ball valve; 4—gas mass flow controller; 5—peristaltic pump; 6—gas–liquid microreactor; 7—solvent bottle; 8—liquid level sensor; 9—the first pH meter; 10—high-voltage constant-current infusion pump; 11—the first pressure sensor; 12—oil bath thermostat; 13—preheating device; 14—the first temperature sensor; 15—the second temperature sensor; 16—sample storage facility; 17—gas–liquid separator; 18—consistent flow valve; 19—waste liquid collection bottle; 20—second pH meter; 21—second pressure sensor; 22—back-pressure valve; and 23—condenser.

First, the gas mass flow controller was used to set the CO_2_ gas flow rate, the peristaltic pump was used to set the water flow rate, the gas and liquid were mixed in real-time at the gas–liquid micromixer, and the pH value in the solvent bottle was monitored in real time using a pH meter so that the gas flow rate can be increased or decreased at any time. The solution’s pH value can be corrected to form a carbonic acid aqueous solution with pH = 5. After that, the temperature of the oil bath thermostat was set to 85 °C, and the reactor containing rock samples was placed in the oil bath thermostat. The flow rate of the high-pressure constant-current infusion pump was set according to the requirements of the four working conditions of the test, and the pressure value of the back-pressure valve was gradually adjusted to form a sample warehouse with a certain pressure and flow rate of carbonic acid water soluble. After the reaction was completed, the gas was separated using the gas–liquid separator. It was directly discharged into the air through the back-pressure valve, and the separated liquid was discharged through the steady flow valve into the waste liquid collection bottle. The pH meter in the bottle continuously monitors the pH of the solution. After the test, the solution was collected in a 50-mL high-density polyethylene plastic bottle before and after the dissolution. The solution’s Ca^2+^ ion content was determined using ICP–OES, and the solution’s conductivity was measured using a CT-2 portable conductivity meter. The rock sample was removed from the reactor, rinsed with deionized water, dried, and reweighed (W2), and its volume was calculated.

## 4. Results and Discussion

The dissolution of 16 carbonate rock specimens in 4 groups of test batches was completed, All four tests used a self-made dissolution instrument conducted in the same laboratory environment. The test results were referenced and reasonable, and the lateral comparative analysis from the macroscopic characteristics, dissolution rate, and hydrochemical composition was expanded.

### 4.1. Macro-Feature Analysis

**Figure 7:**
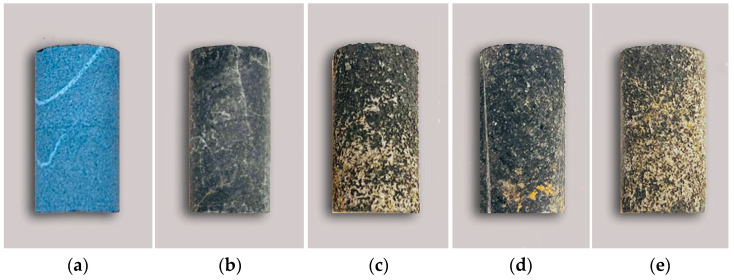
Comparison diagram of rock samples before and after dissolution: (**a**) Original sample; (**b**) Test condition 1; (**c**) Test condition 2; (**d**) Test condition 3; (**e**) Test condition 4.

Figure 7 shows a comparison chart of rock samples before and after acid etching. The dissolution test can simulate the dissolution reaction process. The rock samples in four groups of tests have varying degrees of damage among which the overall shape of the working condition 4 changes noticeably (Figure 7e), and the original mineral surface fluctuates. Noticeable dissolution grooves and grooves appear, accompanied by secondary mineral formation and attachment to the rock surface. The solution corrodes the sample surface strongly under this test condition. However, the overall shape of the working condition was unchanged (Figure 7b), the mineral surface was comparatively smooth, and the acid dissolution of the solution to the sample was weak.

### 4.2. Analysis of Dissolution Rate Change

**Figure 8:**
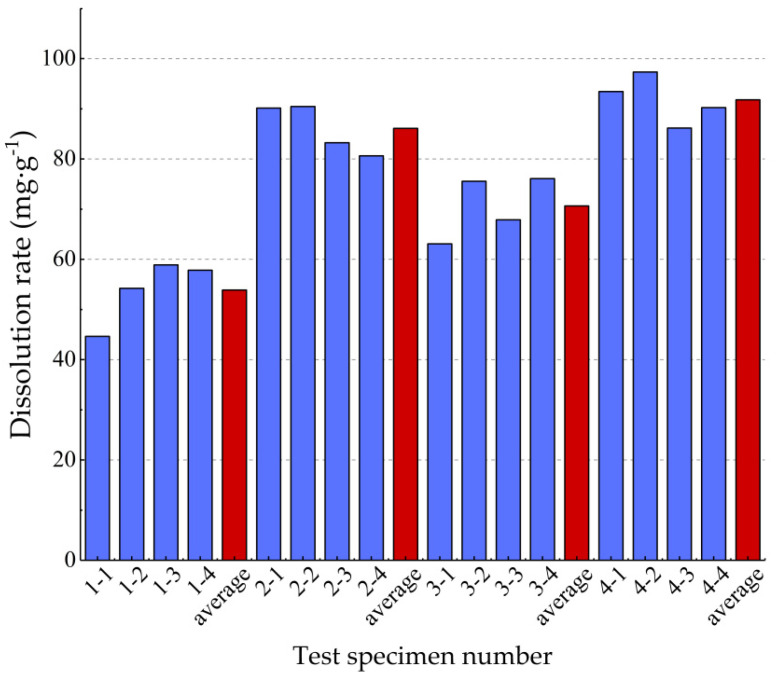
The dissolution rate of 16 carbonate rock specimens.

When the sample was weighed before and after the dissolution, all of these were repeated three times and averaged. Before and after the dissolution was obtained, the rock sample weight was W1 and W2. The dissolution amount is the weight difference (W1–W2) between the rock sample before and after dissolution. The dissolution amount to mass before test ratio is the unit mass dissolution amount, and the dissolution rate = dissolution amount (mg)/weight before dissolution (g). Table 4 shows the dissolution rates of 16 carbonate rock specimens in 4 working conditions groups. Figure 8 shows that the mean dissolution rate in working condition 4 is 91.80 mg/g and 53.89 mg/g in working condition 1, with significant differences in dissolution rates.

#### 4.2.1. Dissolution Characteristics under Different Hydrodynamic Pressures

**Figure 9:**
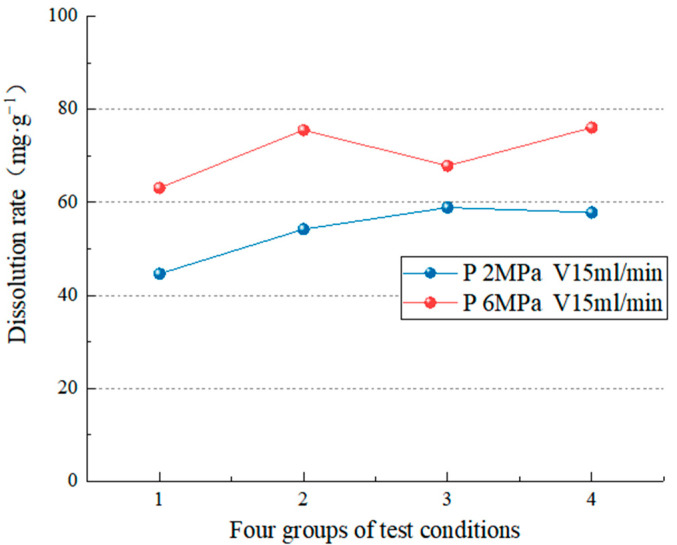
Comparison of the dissolution rate at a water flow velocity of 15 mL/min.

**Figure 10:**
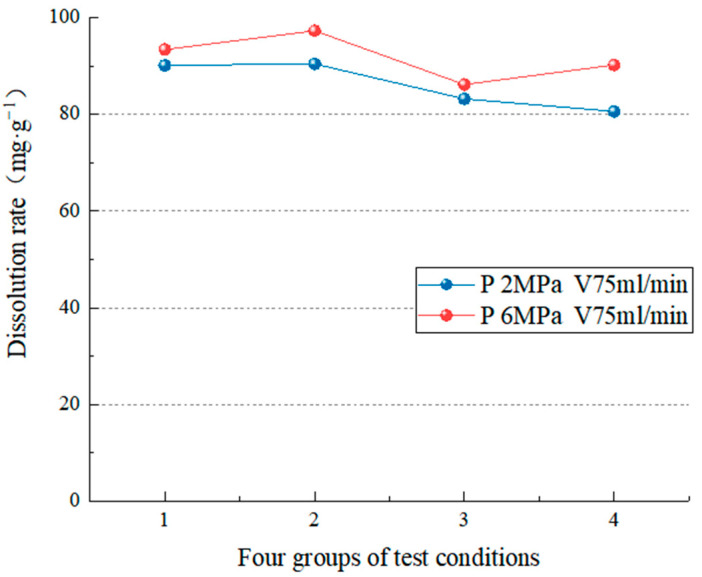
Comparison of the dissolution rate at a water flow velocity of 75 mL/min.

Two groups of test data under different pressure conditions were analyzed with the same velocity of dissolution solution. Figure 9 displays that when the flow rate is 15 mL/min, significantly increasing the pressure influences the dissolution rate (the average dissolution change is 16.76 mg/g). Carbonate rocks dissolved quickly in the early stages of the experiment, the amount of dissolution increased rapidly, and the dissolved minerals were carried away by the current. As dissolution time passes, the surface soluble minerals decrease, and the insoluble or insoluble mineral particles remaining on the rock surface form a film, causing the dissolution rate to decrease. The higher the hydrodynamic pressure, the stronger the pressure will break and erode the film on the surface of the rock sample, controlling the dissolution rate. As a result, as the carbonic acid aqueous solution is continuously added, the dissolved CaCO_3_ and remaining insoluble substances will be continuously taken away, causing the dissolution to worsen. Because of the low velocity, hydrodynamic pressure erosion of the rock sample surface becomes the factor that controls the erosion rate. At this point, pressure will aggravate the degree of carbonate erosion; that is, pressure controls the erosion rate. Figure 10 shows that when the flow rate is 75 mL/min, there is no discernible difference in dissolution rate between 2 MPa and 6 MPa (the average dissolution change is 5.68 mg/g). However, the total dissolution rate at 6 MPa is slightly higher. Because the hydrodynamic pressure has a weaker effect on rock sample breaking and erosion when the solution speed is higher and the solution speed controls the dissolution rate, the dissolution rate of test blocks under the two conditions is relatively close. The test results show that the hydrodynamic pressure is proportional to the dissolution rate regardless of the flow rate. The hydrodynamic pressure significantly influences the dissolution rate when there is a low flow rate.

#### 4.2.2. Dissolution Characteristics under Different Water Flow Velocities

**Figure 11:**
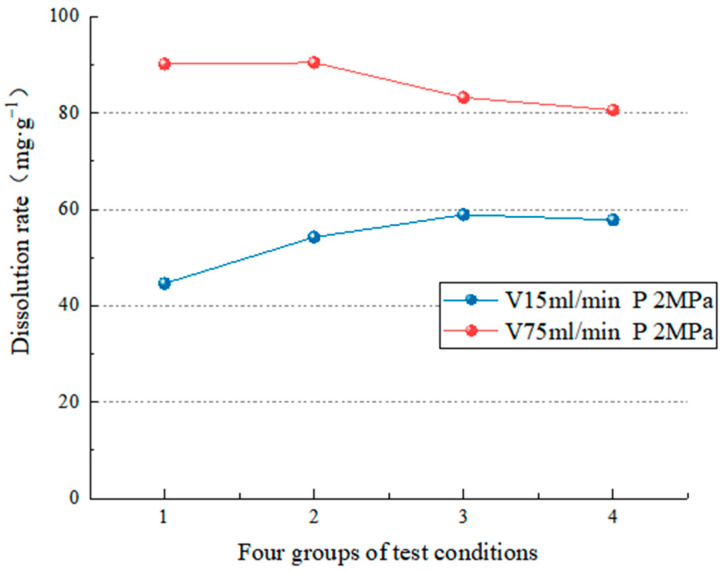
Comparison of the dissolution rate at a pressure of 2 MPa.

**Figure 12:**
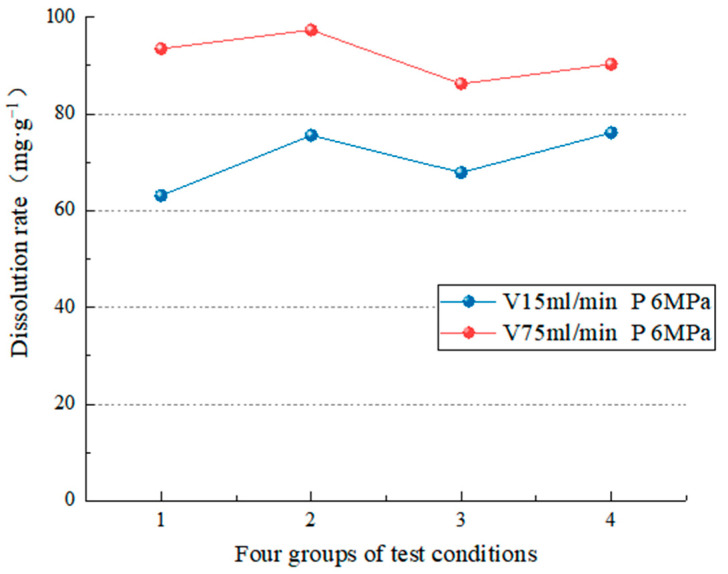
Comparison of the dissolution rate at a pressure of 6 MPa.

Two groups of experimental data with different flow rates were analyzed under the same hydrodynamic pressure. Figure 11 and Figure 12 show that when the hydrodynamic pressure is 2 or 6 MPa, the dissolution rate at a 75-mL/min flow rate is more significant than that at a flow rate of 15 mL/min. This is because the movement of the aqueous solution accelerates the convection and diffusion of ions in the solution, as well as the dissolution rate of minerals, which causes the dissolution rate of minerals to increase with the increase in flow rate; that is, the higher the flow rate, the faster the dissolution rate of salt rock specimens. According to Liu et al.’s diffusion boundary layer theory [56], the higher the velocity, the thinner the diffusion boundary layer of carbonate rocks in the water, and the higher the erosion rate, making carbonate rock erosion more likely. On the other hand, the slower the flow rate, the slower the erosion rate, and the less favorable the environment for erosion.

Figure 11 and Figure 12 show that when the dynamic water pressure is low (2 MPa), the change in water flow velocity significantly influences the dissolution rate (the average dissolution change is 32.23 mg/g). On the other hand, when the dynamic water pressure is high (6 MPa), the influence of water flow velocity on the dissolution speed is small (the average dissolution change is 21.15 mg/g). The test results show that the water flow velocity is proportional to the dissolution rate regardless of dynamic water pressure, and the effect of flow velocity on the dissolution speed is more apparent when the dynamic water pressure is low.

### 4.3. Analysis of the Chemical Composition of Water

#### 4.3.1. The Change Rule of pH Value

**Figure 13:**
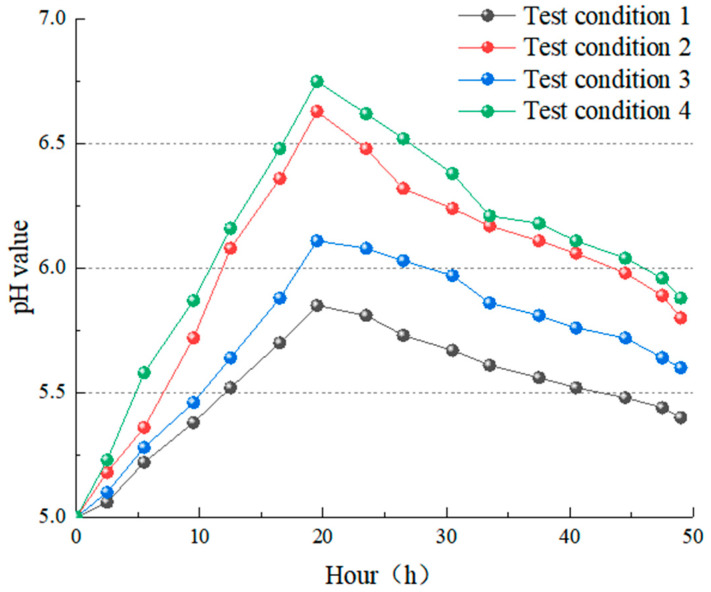
The curve of change of pH value during the test.

The pH value before and after dissolution can be observed at any point during the test. The pH value before the dissolution reaction represents the pH value after the gas–liquid reaction. On the other hand, the pH value after reaction represents the pH value of the liquid flowing out after dissolution, and the concentration index of ion components influences its water change. It was recorded twice a day—2.5 h and 5 h after the start of the test—for 7 days, and the final test was conducted at the end, a total of 16 times. Figure 13 shows the change characteristics of the pH value of the solution with time under four working conditions.

**Figure 14:**
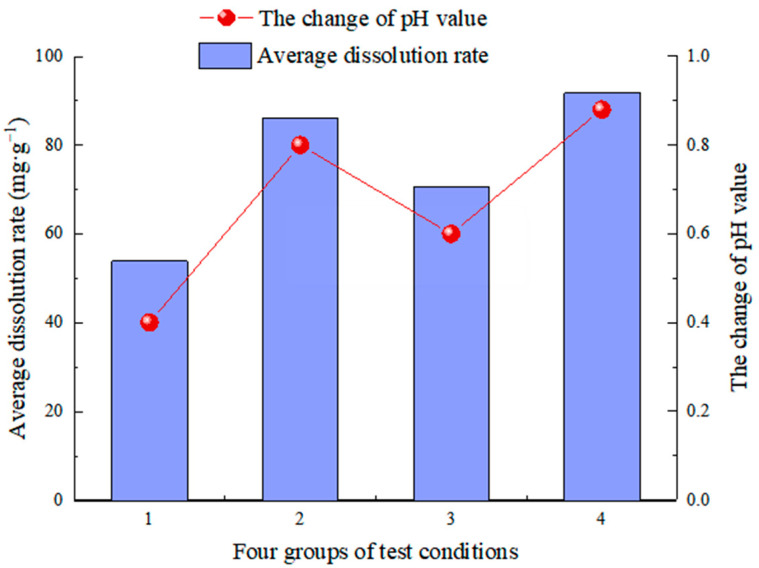
The trend of pH value before and after dissolution.

The pH value of each group in the experiment generally followed the rule of “first increasing and then decreasing, tending to be stable with a little fluctuation” because the pH value of the solution was the result of the combined action of limestone dissolution and carbon dioxide dissolution in the atmosphere. The specimen was relatively dry at the start of the test, and the surface dissolved quickly after being immersed in the solution. Calcium ions were quickly and easily dissolved in the solution during the dissolution process. The limestone dissolution rate was much higher than that of carbon dioxide gas, which consumed many H^+^ ions in the solution. Carbonate rocks took longer to dissolve in the carbonic acid solution, and the amount of H^+^ consumed by the CO_2_ and water reaction was more significant than the amount of HCO_3_^−^ generated. The decrease in H^+^ caused the pH value to rise. After a certain period of dissolution, most of the soluble parts of carbonate rocks had been dissolved, and the dissolution rate had decreased. With the supply of carbonated water in the system, the pH gradually decreased and reached a dynamic equilibrium with the dissolution of carbon dioxide gas, tending to be between 5.40 and 5.88. The pH values of the solution before and after dissolution are +0.40, +0.80, +0.60, and +0.88, as shown in Figure 14. The reaction is more intense in the test batches with a high dissolution rate, and its H^+^ decreases more. Therefore, the pH value increases more, and the changing trend is directly proportional to the change in dissolution rate in the four groups.

#### 4.3.2. The Change Law of Electrical Conductivity

**Figure 15:**
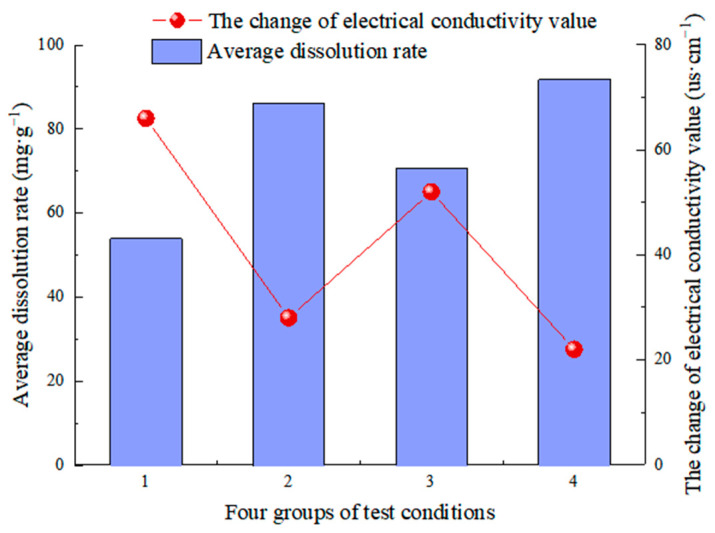
The trend of electrical conductivity value before and after dissolution.

The conductivity of a solution is measured using the CT-2 pen conductivity meter before and after dissolution, which can indicate the ion content of the solution. The conductivity EC of four groups of corrosive solutions increased over time, as shown in Table 5. CO_2_ reacts with water to produce HCO_3_^−^ and ionize Ca^2+^, according to the dissolution reaction formula of carbonate rocks. More ions are precipitated as a result of the reaction. The higher the conductivity value, the more ions in the solution, and vice versa. The changes in conductivity values before and after dissolution are +66 µs/cm, +28 µs/cm, +52 µs/cm, and +22 µs/cm in the four groups of tests, respectively, and the changing trend is inversely proportional to the change in dissolution rate in the four groups of tests (Figure 15). Because of the low flow rate and low hydrodynamic pressure, the longer the reaction solution is in contact with the rock sample surface, the more minerals are dissolved from the fracture surface.

#### 4.3.3. Change Rule of Ca^2+^ Concentration

ICP–OES was used to test the solution before and after dissolution by the American Varian 710-es instrument. The Ca^2+^ ion content was analyzed, another essential index reflecting the contact dissolution performance of the specimen, showing the chemical composition changes of the solution under different working conditions, including Table 6, under the same hydrodynamic pressure. The lower the flow rate, the more significant the change (ΔC) of Ca^2+^ ion concentration after the reaction. Under the same flow rate, the smaller the hydrodynamic pressure, the more significant the change (ΔC) of Ca^2+^ concentration after the reaction. Simultaneously, the lower the flow rate and the lower the hydrodynamic pressure, the longer the contact time between the reaction solution and the rock sample surface will become. Therefore, more minerals will be dissolved from the fracture surface; so the more Ca^2+^ that enters the solution, the more significant the change of Ca^2+^ concentration (ΔC) before and after the reaction, which is the same as the changing trend of conductivity.

## 5. Summary and Conclusions

The self-developed carbonate dissolution test equipment was used in this study to conduct dynamic dissolution tests of carbonate rocks at different temperatures, flow rates, and hydrodynamic pressures. The dissolution effect and development law of carbonate rocks under complex conditions were discussed. The following are the main conclusions:

1. Self-designed and manufactured a set of carbonate rock hydrodynamic pressure dissolution test equipment simulating multifactor coupling conditions, which outperforms the previously closed dissolution test equipment. First, the smaller gas–liquid mixing microreactor was used for the first time in dissolution equipment. The micro-mixing technology ensured rapid and complete mixing of CO_2_ and aqueous solution. Second, the exact configuration of carbonic acid solutions with different concentrations was completed online in real time, ensuring safe, continuous, stable, and efficient gas–liquid mixing, breaking the limitation of pH parameter selection and the stability of previous test instruments, and significantly reducing the volume of carbonated water preparation device [26,37,50,51]. Next, the test system employs computer control software to control and set related devices and parameters, which reduces the difficulty of using the instrument, ensures numerical value accuracy, and enables intuitive observation. Third, the pressure and hydrodynamic device realize the combined regulation of solution dynamic water pressure and speed, raises and stabilizes the liquid pressure via the back pressure valve and adjusts the flow rate via the high-pressure constant-current infusion pump to ensure that the solution can complete the flow rate adjustment at different pressures. It breaks through the previous test hydrodynamic system that cannot be regulated simultaneously with the pressure system [26,51,52,53,55];
2. The test simulates carbonate rock’s dissolution effect in different environmental factors (such as water chemical conditions, temperature conditions, dynamic water pressure conditions, and water flow speed conditions). In the analysis of the carbonate rock dissolution rate, the dissolution amount of the carbonate rock is directly proportional to the water flow speed [37], the dynamic water pressure [19,25,39,40], and the influence of the flow rate on the dissolution rate is more significant than the dynamic water pressure [38]. When the water flow rate is high (75 mL/min), the change in hydrodynamic pressure slightly impacts the dissolution rate (the average dissolution rate is 5.68 mg/g). However, when the flow rate is low (15 mL/min), the hydrodynamic pressure change significantly (the average dissolution rate is 16.76 mg/g). In addition, when the hydrodynamic pressure is low (2 MPa), the change in water velocity significantly impacts the dissolution rate (the average dissolution rate is 32.23 mg/g). On the other hand, when the hydrodynamic pressure is high (6 MPa), the change in water velocity has little impact (the average dissolution rate is 21.15 mg/g). To realize the coupling of water flow speed and dynamic water pressure, it breaks through the coupling of two factors that cannot be achieved by previous test, and also lack of research of the dissolution effect and the development law under the coupling action;
3. CO_2_ aqueous solution significantly influences limestone dissolution and is the most important carbonate dissolution medium in the near-surface supergene condition. Following dissolution, visible dissolution grooves and grooves appear on the surface of rock samples, along with new calcium carbonate deposits. The changes in the solution’s pH value, conductivity value, and Ca^2+^ ion concentration show the dissolution rate and degree. The values after dissolution are greater than those before the test. The pH values were changed at +0.40, +0.80, +0.60, and +0.88, which are directly proportional to the change in the dissolution rate [57,58]. The conductivity values were +66 µs/cm, +28 µs/cm, +52 µs/cm and +22 µs/cm, and the Ca^2+^ ion content was +13.05, +6.48 and +10.25, respectively, which were inversely proportional to the change in dissolution rate [55]. The pH value generally follows the law of “first increasing and then decreasing, tending to be stable with a little fluctuation” during the test, which can effectively reflect the dissolution process of limestone under actual conditions.

The experimental study shows carbonate dissolution is proportional to the water flow velocity and dynamic water pressure close to the ground surface. The runoff change of groundwater near the surface obviously influences the dissolution rate. In addition, the karst develops more in the groundwater runoff than in the groundwater’s recharge and discharge areas. In the case of a slow groundwater flow rate, the dynamic water pressure greatly influences the dissolution rate, and the karst develops more. Therefore, its study has important theoretical significance and engineering application value as it provides technical means and guarantees for the early identification, karst development, and safety evaluation of karst geological disasters.

In subsequent studies, the author will present the influence and change of the law of the coupling effect of water flow velocity change, hydrodynamic pressure change, thermal–hydraulic–chemical, and other factors on the dissolution effect in a subsequent paper.

## Figures and Tables

**Table 1:** XRD analyses of carbonate rocks.

Mineral Composition	Mineral Content (%)
Quartz	1.5
Calcite	96.5
Else	2.0

Note: Other substances mainly include weak crystallization or amorphous substances.

**Table 2:** Main chemical composition content of the rock sample and LS (wt%).

Chemical Components	wt (%)
SiO_2_	0.532
CaO	55.41
Fe_2_O_3_	0.206
CO_2_	43.54
MgO	0.050
Al_2_O_3_	0.053
SrO	0.052
Cl	0.040
Loss on ignition	0.117

**Table 3:** Dissolution conditions under Four groups of tests.

Test Batch Number	pH Value	Pressure (MPa)	Temperature (°C)	Current Speed (mL·min^–1^)	Duration (d)
1	5	2	85	15	7
2	5	2	85	75	7
3	5	6	85	15	7
4	5	6	85	75	7

**Table 4:** The dissolution rate of carbonate rocks under four groups of test conditions.

Test Batch Number	Pressure (MPa)	Current Speed (mL·min^–1^)	Test Specimen Number	Before Dissolution Weight (g)	After Dissolution Weight (g)	Dissolution (g)	Dissolution Rate (mg·g^−1^)
1	2	15	1-1	8.29	7.92	0.37	44.632
			1-2	8.30	7.85	0.45	54.217
			1-3	8.32	7.83	0.49	58.894
			1-4	8.30	7.82	0.48	57.821
			average value	8.30	7.86	0.45	53.89
2	2	75	2-1	8.32	7.57	0.63	90.144
			2-2	8.29	7.54	0.75	90.470
			2-3	8.29	7.60	0.69	83.233
			2-4	8.31	7.64	0.67	80.626
			average value	8.30	7.59	0.69	86.12
3	6	15	3-1	8.40	7.87	0.53	63.095
			3-2	8.47	7.83	0.64	75.560
			3-3	8.36	8.01	0.35	67.866
			3-4	8.28	7.65	0.63	76.087
			average value	8.38	7.84	0.54	70.65
4	6	75	4-1	8.24	7.47	0.77	93.447
			4-2	8.22	7.42	0.80	97.324
			4-3	8.27	7.64	0.63	86.179
			4-4	8.20	7.46	0.74	90.243
			average value	8.23	7.50	0.74	91.80

**Table 5:** Solution conductance data under four groups of test conditions.

Test Batch Number	Pressure (MPa)	Current Speed (mL·min^–1^)	Before Dissolution (µs·cm^–1^)	After Dissolution (µs·cm^–1^)	Variety (µs·cm^–1^)
1	2	15	266	332	+66
2	2	75	284	312	+28
3	6	15	261	317	+52
4	6	75	269	291	+22

**Table 6:** Solution Ca^2+^ concentration data under four groups of test conditions.

Test Batch Number	Pressure (MPa)	Current Speed (mL·min^−1^)	Before Dissolution Ca^2+^ Ion Concentration (mg·L^−1^)	After Dissolution Ca^2+^ Ion Concentration (mg·L^−1^)	The ΔCa^2+^ Ion Concentration Was Precipitated (mg·L^−1^)
1	2	15	17.68	30.73	+13.05
2	2	75	29.02	35.50	+6.48
3	6	15	27.24	37.49	+10.25
4	6	75	27.00	33.34	+6.34

## Data Availability

The data used to support the findings of this study are available from the corresponding author upon request.
